# Editorial: Microbial Communities and Metabolisms Involved in the Degradation of Cellular and Extracellular Organic Biopolymers

**DOI:** 10.3389/fmicb.2021.802619

**Published:** 2022-01-04

**Authors:** S. Emil Ruff

**Affiliations:** Marine Biological Laboratory, Woods Hole, MA, United States

**Keywords:** macromolecule, necromass, heterotrophic microorganism, protein, polysaccharide, carbohydrate, nucleic acid, lipid

Most organic matter on Earth occurs in the form of macromolecules and complex biopolymers, which include the building blocks of every organism. Plant, animal, fungal, and microbial cells largely consist of macromolecules belonging to four compound classes: proteins, polysaccharides, nucleic acids, and lipids (Figure 1). The percentage of these compounds per dry weight can vary greatly between lineages, but also between individuals of the same species or developmental stages of the same organism. Living and lysing cells release a substantial quantity and variety of macromolecules to the environment. These compounds often contain nitrogen, phosphorus, and sulfur, in addition to carbon, and are thus ideal food sources for heterotrophic organisms. Although the degradation of biopolymers and macromolecules has received considerable attention, many knowledge gaps remain, particularly in very complex ecosystems such as soils and sediments.

**Figure 1:**
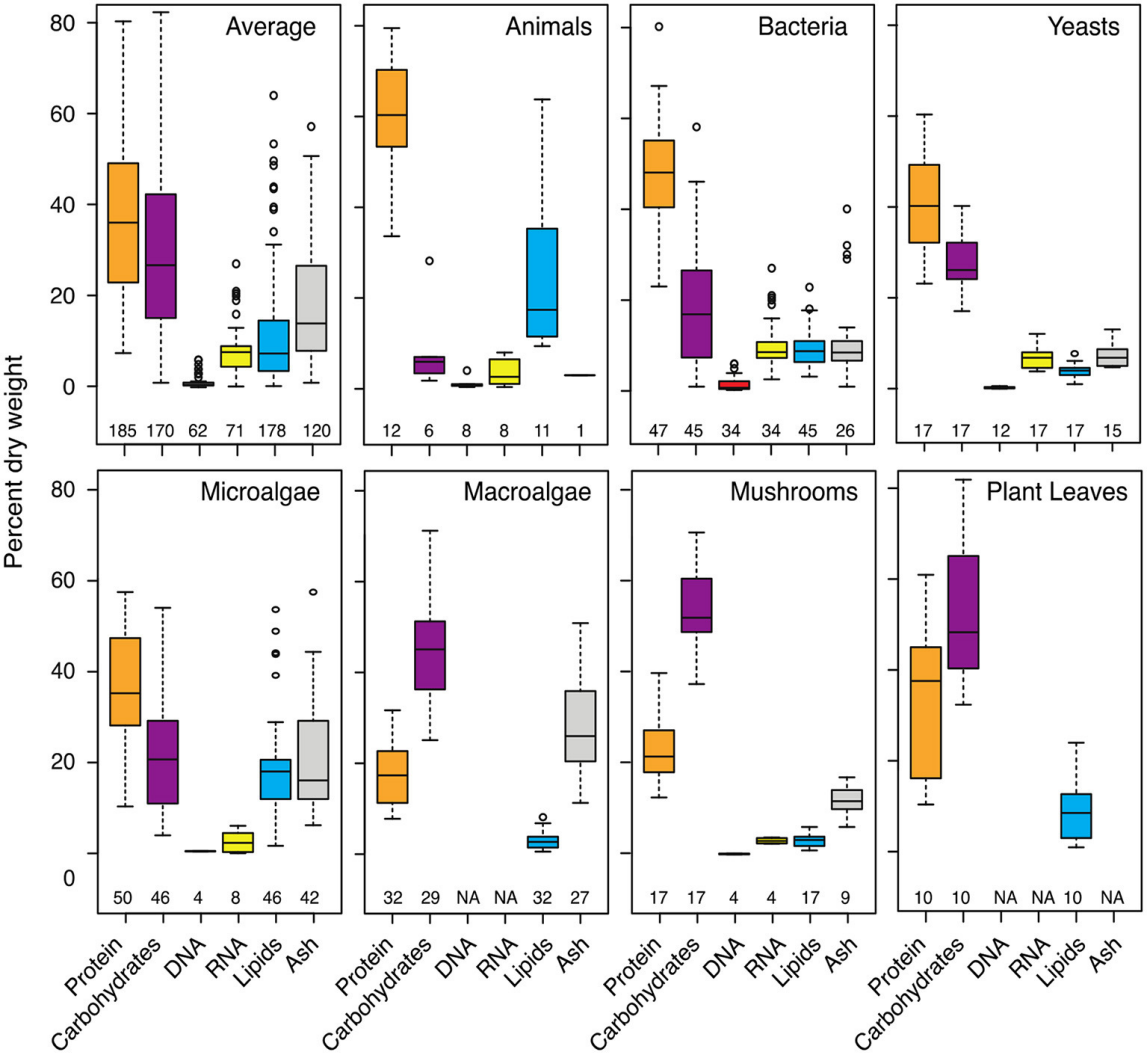
Abundance of major classes of cellular macromolecules in different organisms. The macromolecular composition of cells and organisms can vary greatly between lineages, between species, and even between different body parts or developmental stages of the same organism. Proteins and carbohydrates (mainly polysaccharides) constitute most of the biomass of a generic cell averaged across all included organisms. The abundance and importance of proteins or polysaccharides or other macromolecules as a source of energy, carbon, and nutrition, however, greatly differs between ecosystems depending on its community. The values are taken from studies listed in Supplementary Table 1. The number of included samples is given below each boxplot. “Average” percentages were calculated from all shown organisms and are subject to change with additional data.

The articles in this collection and other recent studies shed light on the metabolic versatility and the distinct ecological niches of biopolymer-degrading organisms and communities, and yield new insight into metabolisms, syntrophic interactions, and biogeochemical constraints of biopolymer degradation.

A major question in the cycling of organic matter concerns the response of heterotrophic soil microbes to long-term soil warming. Soils constitute an important carbon sink, and carbon storage in soils is vulnerable to microbial degradation with increasing climate warming. For instance, after 30 years of experimental warming, carbon stocks in a temperate forest were observed to be reduced by 30% in the heated plots relative to the controls (Roy Chowdhury et al.). In addition, warming caused enhanced gene expression of certain carbohydrate active enzymes (CAZymes) and increased abundances of enzymes related to polysaccharide and lipid metabolisms (Roy Chowdhury et al.). The effect of long-term warming on community gene expression indicates a higher carbohydrate degrading potential of soil bacteria that possibly accelerates a self-reinforcing carbon cycle-temperature feedback in a warming climate.

The genus Flavobacterium is known for its functional role in mediating polysaccharide degradation in soil and is often found in root microbiomes. Members within this genus are characterized by the capacity to metabolize a broad spectrum of complex carbohydrates and a unique gliding motility mechanism (Gavriilidou et al., 2020). It was shown that certain flavobacterial strains exhibit gliding motility on agar plates with monomeric pectin and other polysaccharides such as microcrystalline cellulose. However, only polymeric pectin, a component of plant cell walls, enhanced colony expansion on solid surfaces in a dose- and substrate-dependent manner (Kraut-Cohen et al.). Proteomic and gene expression analyses further revealed significant induction of carbohydrate metabolism related proteins when flavobacteria were grown on pectin, suggesting that pectin may facilitate flavobacterial expansion on plant surfaces in addition to serving as an essential carbon source.

A large proportion of pectin degradation, as well as of polysaccharides and polymeric organic matter degradation occurs in the periplasm. Many heterotrophic microorganisms are capable of producing extracellular enzymes to hydrolyze organic macromolecules outside the cell, the mono-/ and oligomers can then be transported across the cell membrane and metabolized inside the cell (Pérez Castro et al., 2021). Therefore, extracellular enzyme activity may be a good indicator of the fate of organic carbon in nature. The seafloor of the Baltic Sea, for example, contained active extracellular peptidases, alkaline phosphatase, and β-glucosidases in old sediments at depths of up to 54 m below the seafloor (Schmidt et al.). These extracellular enzymes appear to be extremely stable, retaining on average 50% of their activity after autoclaving for an hour. Also, these enzymes seem to have extraordinarily long lifetimes, reaching the order of hundreds to thousands of hours (Schmidt et al.). Together, these results lend empirical support that a population of subsurface microbes might be able to persist in this hidden ecosystem by using extracellular enzymes to slowly metabolize old and highly degraded organic carbon.

Extracellular hydrolysis is also widespread in the surface ocean and can occur by two distinct mechanisms: “selfish uptake,” in which initial hydrolysis is coupled to transport of large polysaccharide fragments into the periplasmic space of bacteria, with little to no loss of hydrolysis products to the external environment, and “external hydrolysis,” in which low molecular weight hydrolysis products are produced in the external environment (Reintjes et al.). Although all polysaccharides investigated by the authors were found to be degraded *via* both mechanisms, it appears as if the algal polysaccharide fucoidan was mainly consumed selfishly. Overall, a diverse set of bacterial populations were reported to be important for the recycling of polymeric organic matter derived from algal blooms (Reintjes et al.).

One of those marine polymer degraders is the newly described flavobacterial strain Maribacter dokdonensis 62-1 (Wolter et al.). This organism harbors a diverse array of CAZymes including polysaccharide lyases that allow substantial growth with alginate as sole carbon source, and simultaneous utilization of mannuronate and guluronate. A comparison with related Maribacter and Zobellia strains indicated specialization to certain polysaccharides, a finding supported by a recent study showing that certain Verrucomicrobiota have distinct polysaccharide preferences (Orellana et al., 2021). In sum, the genus Maribacter contains versatile polysaccharide degraders, with implications for biogeochemical cycles, niche specialization, and bacteria-algae interactions in the oceans.

Microbes have the unique ability to break down the complex polysaccharides that make up the bulk of organic matter, yet these organisms often do not perform in solitude (D'Souza et al., 2021). Recent advances in microbial ecology suggest that polysaccharide persistence can result from non-linear growth dynamics created by the coexistence of alternate degradation strategies, metabolic roles, as well as by ecological interactions between microbes (Sichert and Cordero). These complex degradation strategies and interspecific interactions are shaped by different processes, including the evolution of genetic repertoires, phenotypic heterogeneity, metabolic interactions, and microbial cooperation (Sichert and Cordero). Thus, there is ample opportunity and need to study polymer-bacteria interactions in the context of eco-evolutionary dynamics across divergent systems.

The need for understanding microbial biopolymer degradation goes well-beyond basic science as there are applications of high relevance. Incubation of the biodegradable plastic materials polyhydroxybutyrate (PHB), polybutylene sebacate (PBSe), and polybutylene sebacate co-terephthalate (PBSeT) under different conditions showed that the half-life of each compound differed by orders of magnitude depending on climate and habitat (Lott et al.). The biodegradation performance of the materials revealed the impreciseness to generically term a material “marine biodegradable.” The findings of this study will help to inform the life cycle assessment of bioplastics in the open environment as even biodegradable plastics can have long residence times in nature when they are being metabolized very slowly.

Investigating biopolymer-degrading heterotrophic organisms and communities will improve our understanding of the biodiversity and function of ecosystems and elucidate the role that these microbes play in the cycling of organic matter. The findings reported in this collection are relevant and exciting in light of the immense diversity of biopolymers that occur in nature and their increasing importance as solutions for a sustainable future.

## Author Contributions

SER: collected, reanalyzed, and visualized data for Figure 1 and wrote the manuscript.

## Conflict of Interest

The author declares that the research was conducted in the absence of any commercial or financial relationships that could be construed as a potential conflict of interest.

## Publisher's Note

All claims expressed in this article are solely those of the authors and do not necessarily represent those of their affiliated organizations, or those of the publisher, the editors and the reviewers. Any product that may be evaluated in this article, or claim that may be made by its manufacturer, is not guaranteed or endorsed by the publisher.
